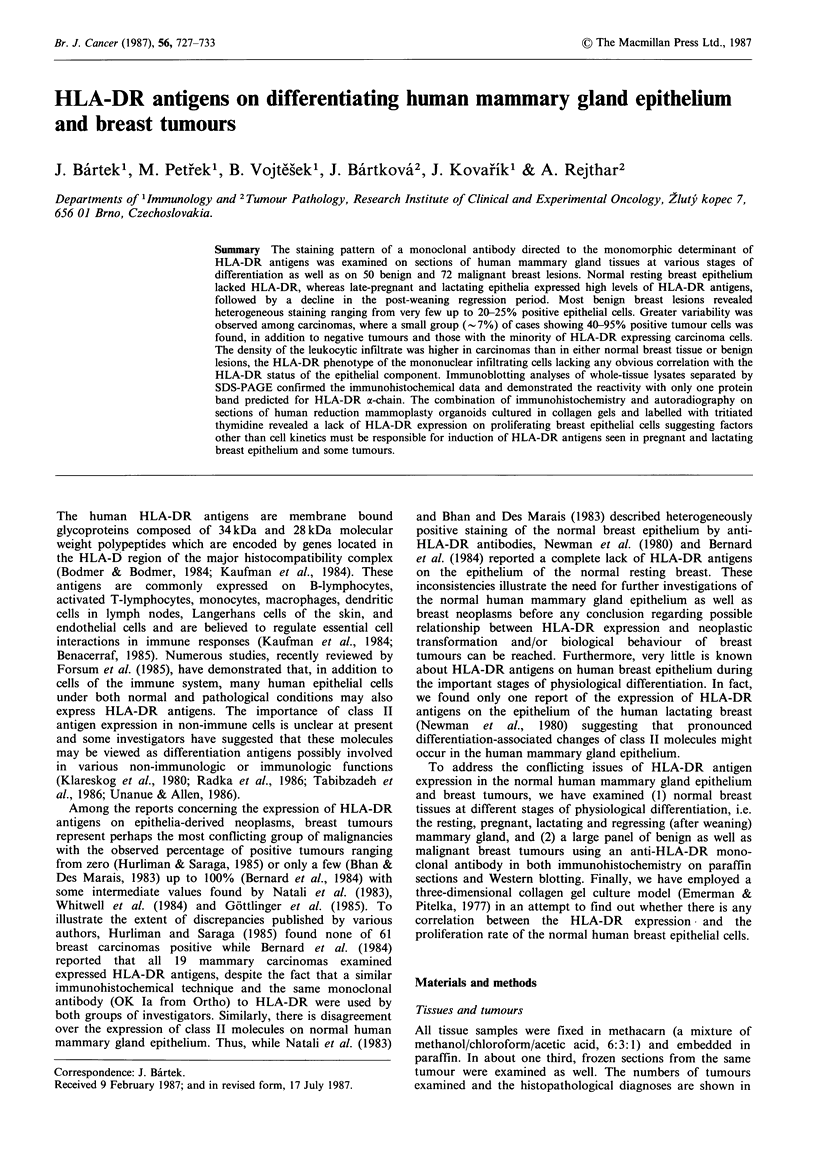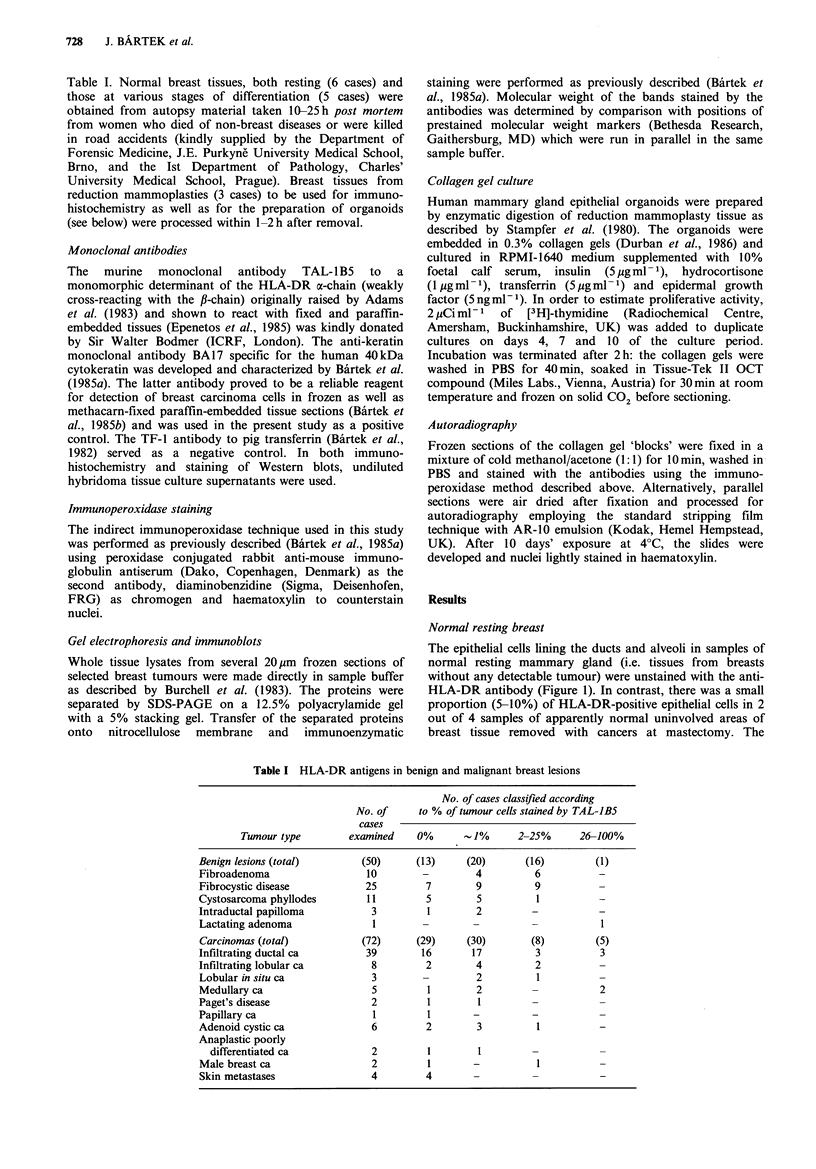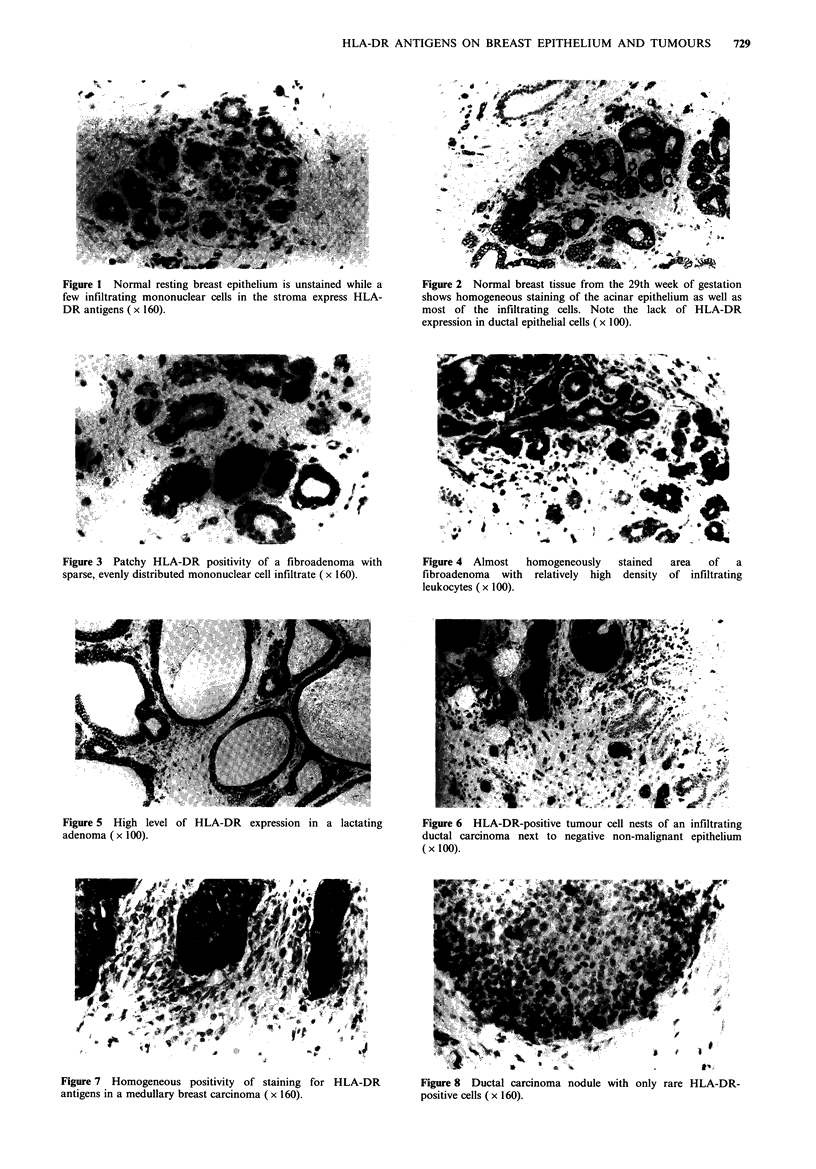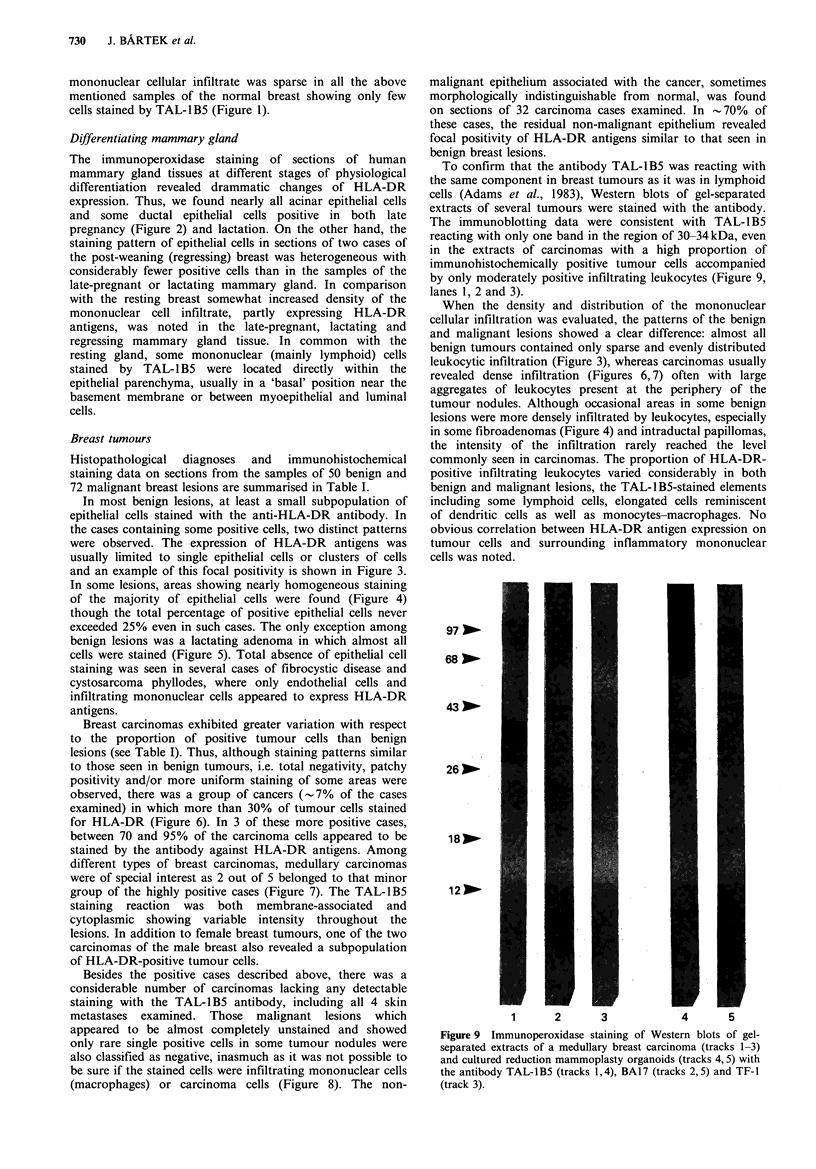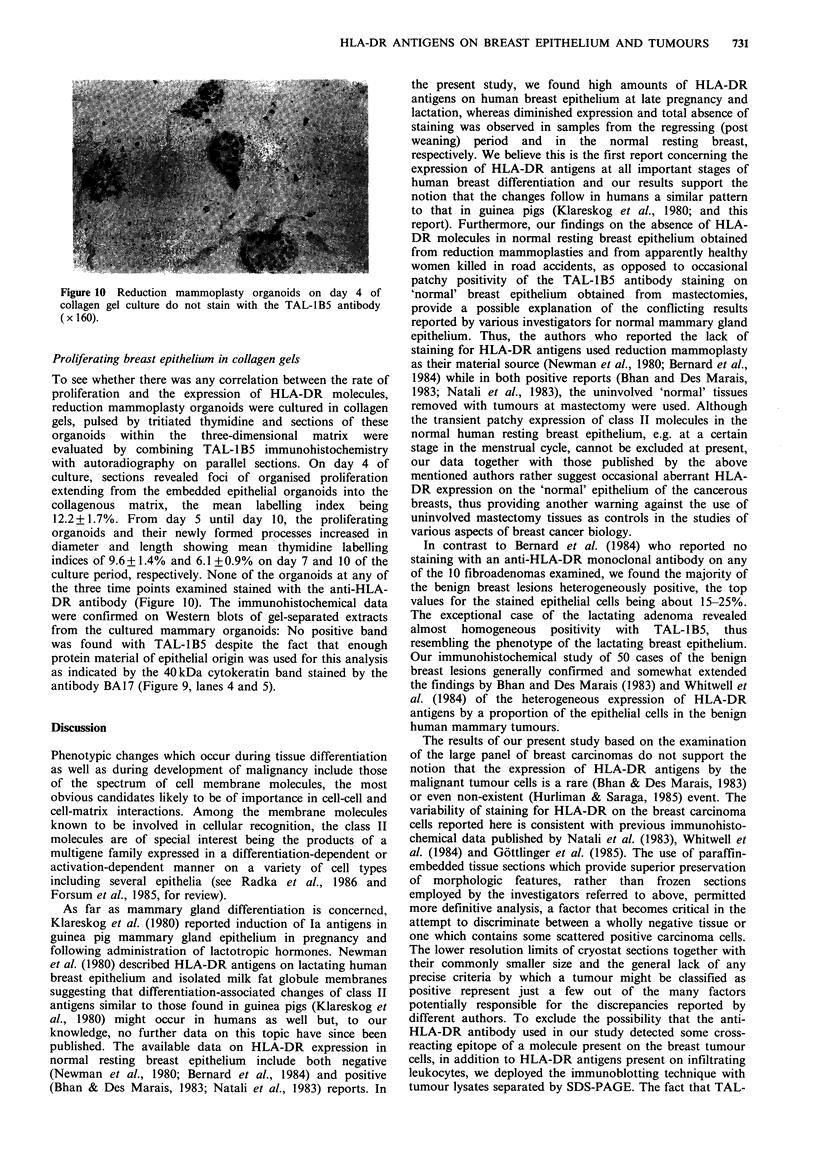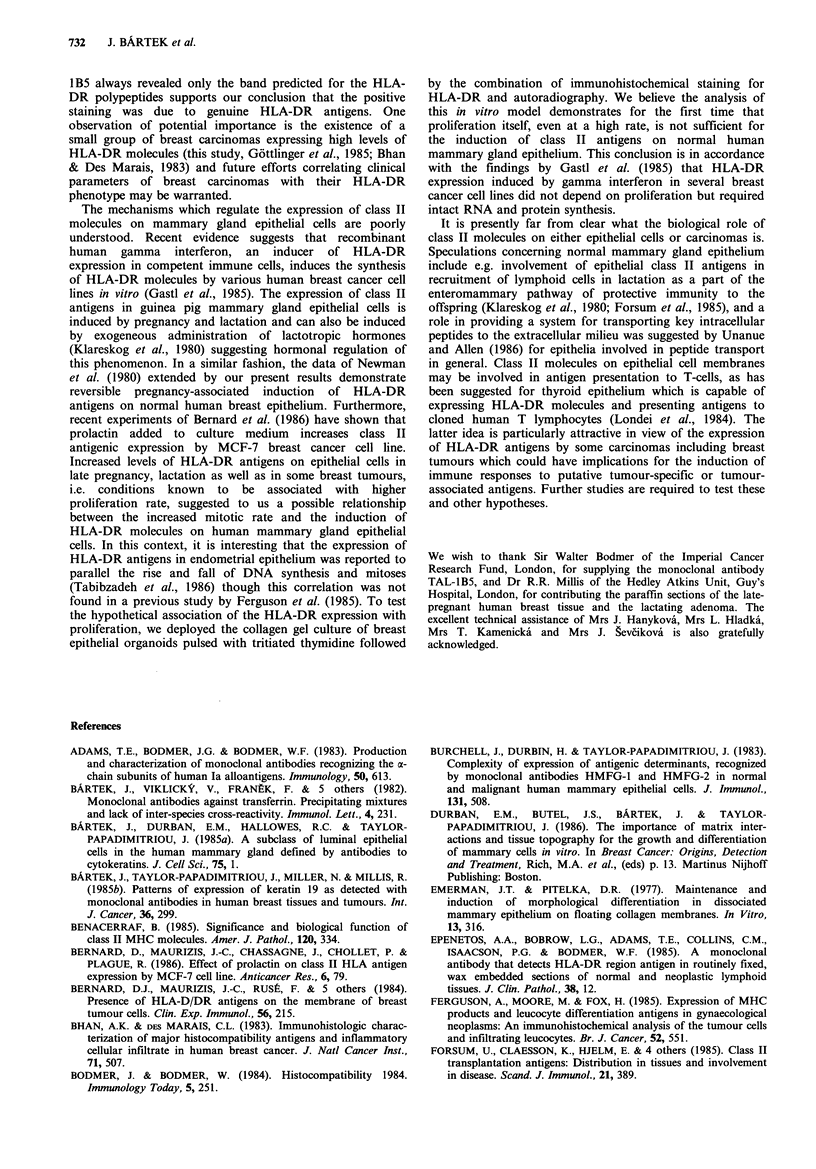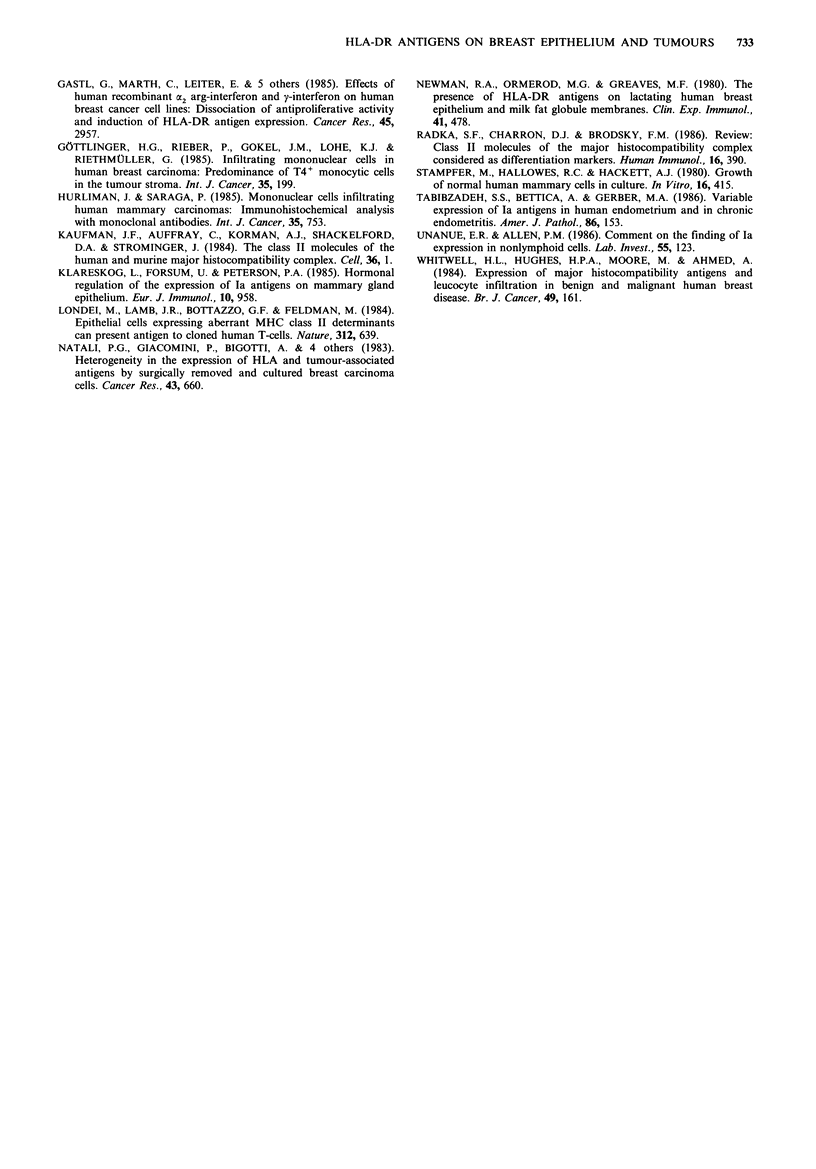# HLA-DR antigens on differentiating human mammary gland epithelium and breast tumours.

**DOI:** 10.1038/bjc.1987.278

**Published:** 1987-12

**Authors:** J. Bártek, M. Petrek, B. Vojtĕsek, J. Bártková, J. Kovarík, A. Rejthar

**Affiliations:** Department of Immunology, Research Institute of Clinical and Experimental Oncology, Brno, Czechoslovakia.

## Abstract

**Images:**


Br. J. Cancer (1987), 56, 727–733 © The Macmillan Press Ltd., 1987

### HLA-DR antigens on differentiating human mammary gland epithelium and breast tumours

J. Bártek[1], M. Petřek[1], B. Vojtěšek[1], J. Bártková[2], J. Kovařík[1] & A. Rejthar[2]

*Departments of [1]Immunology and [2]Tumour Pathology, Research Institute of Clinical and Experimental Oncology, Žlutý kopec 7, 656 01 Brno, Czechoslovakia.*

**Summary** The staining pattern of a monoclonal antibody directed to the monomorphic determinant of HLA-DR antigens was examined on sections of human mammary gland tissues at various stages of differentiation as well as on 50 benign and 72 malignant breast lesions. Normal resting breast epithelium lacked HLA-DR, whereas late-pregnant and lactating epithelia expressed high levels of HLA-DR antigens, followed by a decline in the post-weaning regression period. Most benign breast lesions revealed heterogeneous staining ranging from very few up to 20–25% positive epithelial cells. Greater variability was observed among carcinomas, where a small group (~7%) of cases showing 40–95% positive tumour cells was found, in addition to negative tumours and those with the minority of HLA-DR expressing carcinoma cells. The density of the leukocytic infiltrate was higher in carcinomas than in either normal breast tissue or benign lesions, the HLA-DR phenotype of the mononuclear infiltrating cells lacking any obvious correlation with the HLA-DR status of the epithelial component. Immunoblotting analyses of whole-tissue lysates separated by SDS-PAGE confirmed the immunohistochemical data and demonstrated the reactivity with only one protein band predicted for HLA-DR α-chain. The combination of immunohistochemistry and autoradiography on sections of human reduction mammoplasty organoids cultured in collagen gels and labelled with tritiated thymidine revealed a lack of HLA-DR expression on proliferating breast epithelial cells suggesting factors other than cell kinetics must be responsible for induction of HLA-DR antigens seen in pregnant and lactating breast epithelium and some tumours.

The human HLA-DR antigens are membrane bound glycoproteins composed of 34 kDa and 28 kDa molecular weight polypeptides which are encoded by genes located in the HLA-D region of the major histocompatibility complex (Bodmer & Bodmer, 1984; Kaufman *et al.*, 1984). These antigens are commonly expressed on B-lymphocytes, activated T-lymphocytes, monocytes, macrophages, dendritic cells in lymph nodes, Langerhans cells of the skin, and endothelial cells and are believed to regulate essential cell interactions in immune responses (Kaufman *et al.*, 1984; Benacerraf, 1985). Numerous studies, recently reviewed by Forsum *et al.* (1985), have demonstrated that, in addition to cells of the immune system, many human epithelial cells under both normal and pathological conditions may also express HLA-DR antigens. The importance of class II antigen expression in non-immune cells is unclear at present and some investigators have suggested that these molecules may be viewed as differentiation antigens possibly involved in various non-immunologic or immunologic functions (Klareskog *et al.*, 1980; Radka *et al.*, 1986; Tabibzadeh *et al.*, 1986; Unanue & Allen, 1986).

Among the reports concerning the expression of HLA-DR antigens on epithelia-derived neoplasms, breast tumours represent perhaps the most conflicting group of malignancies with the observed percentage of positive tumours ranging from zero (Hurliman & Saraga, 1985) or only a few (Bhan & Des Marais, 1983) up to 100% (Bernard *et al.*, 1984) with some intermediate values found by Natali *et al.* (1983), Whitwell *et al.* (1984) and Göttlinger *et al.* (1985). To illustrate the extent of discrepancies published by various authors, Hurliman and Saraga (1985) found none of 61 breast carcinomas positive while Bernard *et al.* (1984) reported that all 19 mammary carcinomas examined expressed HLA-DR antigens, despite the fact that a similar immunohistochemical technique and the same monoclonal antibody (OK Ia from Ortho) to HLA-DR were used by both groups of investigators. Similarly, there is disagreement over the expression of class II molecules on normal human mammary gland epithelium. Thus, while Natali *et al.* (1983) and Bhan and Des Marais (1983) described heterogeneously positive staining of the normal breast epithelium by anti-HLA-DR antibodies, Newman *et al.* (1980) and Bernard *et al.* (1984) reported a complete lack of HLA-DR antigens on the epithelium of the normal resting breast. These inconsistencies illustrate the need for further investigations of the normal human mammary gland epithelium as well as breast neoplasms before any conclusion regarding possible relationship between HLA-DR expression and neoplastic transformation and/or biological behaviour of breast tumours can be reached. Furthermore, very little is known about HLA-DR antigens on human breast epithelium during the important stages of physiological differentiation. In fact, we found only one report of the expression of HLA-DR antigens on the epithelium of the human lactating breast (Newman *et al.*, 1980) suggesting that pronounced differentiation-associated changes of class II molecules might occur in the human mammary gland epithelium.

To address the conflicting issues of HLA-DR antigen expression in the normal human mammary gland epithelium and breast tumours, we have examined (1) normal breast tissues at different stages of physiological differentiation, i.e. the resting, pregnant, lactating and regressing (after weaning) mammary gland, and (2) a large panel of benign as well as malignant breast tumours using an anti-HLA-DR monoclonal antibody in both immunohistochemistry on paraffin sections and Western blotting. Finally, we have employed a three-dimensional collagen gel culture model (Emerman & Pitelka, 1977) in an attempt to find out whether there is any correlation between the HLA-DR expression and the proliferation rate of the normal human breast epithelial cells.

### Materials and methods

### *Tissues and tumours*

All tissue samples were fixed in methacarn (a mixture of methanol/chloroform/acetic acid, 6:3:1) and embedded in paraffin. In about one third, frozen sections from the same tumour were examined as well. The numbers of tumours examined and the histopathological diagnoses are shown in

Correspondence: J. Bártek.
Received 9 February 1987; and in revised form, 17 July 1987.

Table I. Normal breast tissues, both resting (6 cases) and those at various stages of differentiation (5 cases) were obtained from autopsy material taken 10–25 h *post mortem* from women who died of non-breast diseases or were killed in road accidents (kindly supplied by the Department of Forensic Medicine, J.E. Purkyně University Medical School, Brno, and the Ist Department of Pathology, Charles' University Medical School, Prague). Breast tissues from reduction mammoplasties (3 cases) to be used for immunohistochemistry as well as for the preparation of organoids (see below) were processed within 1–2 h after removal.

*Monoclonal antibodies*

The murine monoclonal antibody TAL-1B5 to a monomorphic determinant of the HLA-DR α-chain (weakly cross-reacting with the β-chain) originally raised by Adams *et al.* (1983) and shown to react with fixed and paraffin-embedded tissues (Epenetos *et al.*, 1985) was kindly donated by Sir Walter Bodmer (ICRF, London). The anti-keratin monoclonal antibody BA17 specific for the human 40 kDa cytokeratin was developed and characterized by Bártek *et al.* (1985*a*). The latter antibody proved to be a reliable reagent for detection of breast carcinoma cells in frozen as well as methacarn-fixed paraffin-embedded tissue sections (Bártek *et al.*, 1985*b*) and was used in the present study as a positive control. The TF-1 antibody to pig transferrin (Bártek *et al.*, 1982) served as a negative control. In both immunohistochemistry and staining of Western blots, undiluted hybridoma tissue culture supernatants were used.

*Immunoperoxidase staining*

The indirect immunoperoxidase technique used in this study was performed as previously described (Bártek *et al.*, 1985*a*) using peroxidase conjugated rabbit anti-mouse immunoglobulin antiserum (Dako, Copenhagen, Denmark) as the second antibody, diaminobenzidine (Sigma, Deisenhofen, FRG) as chromogen and haematoxylin to counterstain nuclei.

*Gel electrophoresis and immunoblots*

Whole tissue lysates from several 20 μm frozen sections of selected breast tumours were made directly in sample buffer as described by Burchell *et al.* (1983). The proteins were separated by SDS-PAGE on a 12.5% polyacrylamide gel with a 5% stacking gel. Transfer of the separated proteins onto nitrocellulose membrane and immunoenzymatic staining were performed as previously described (Bártek *et al.*, 1985*a*). Molecular weight of the bands stained by the antibodies was determined by comparison with positions of prestained molecular weight markers (Bethesda Research, Gaithersburg, MD) which were run in parallel in the same sample buffer.

*Collagen gel culture*

Human mammary gland epithelial organoids were prepared by enzymatic digestion of reduction mammoplasty tissue as described by Stampfer *et al.* (1980). The organoids were embedded in 0.3% collagen gels (Durban *et al.*, 1986) and cultured in RPMI-1640 medium supplemented with 10% foetal calf serum, insulin (5 $\mu g\,ml^{-1}$), hydrocortisone (1 $\mu g\,ml^{-1}$), transferrin (5 $\mu g\,ml^{-1}$) and epidermal growth factor (5 $ng\,ml^{-1}$). In order to estimate proliferative activity, 2 $\mu Ci\,ml^{-1}$ of [$^3$H]-thymidine (Radiochemical Centre, Amersham, Buckinhamshire, UK) was added to duplicate cultures on days 4, 7 and 10 of the culture period. Incubation was terminated after 2 h: the collagen gels were washed in PBS for 40 min, soaked in Tissue-Tek II OCT compound (Miles Labs., Vienna, Austria) for 30 min at room temperature and frozen on solid $CO_2$ before sectioning.

*Autoradiography*

Frozen sections of the collagen gel 'blocks' were fixed in a mixture of cold methanol/acetone (1:1) for 10 min, washed in PBS and stained with the antibodies using the immunoperoxidase method described above. Alternatively, parallel sections were air dried after fixation and processed for autoradiography employing the standard stripping film technique with AR-10 emulsion (Kodak, Hemel Hempstead, UK). After 10 days' exposure at 4°C, the slides were developed and nuclei lightly stained in haematoxylin.

### Results

*Normal resting breast*

The epithelial cells lining the ducts and alveoli in samples of normal resting mammary gland (i.e. tissues from breasts without any detectable tumour) were unstained with the anti-HLA-DR antibody (Figure 1). In contrast, there was a small proportion (5–10%) of HLA-DR-positive epithelial cells in 2 out of 4 samples of apparently normal uninvolved areas of breast tissue removed with cancers at mastectomy. The

**Table I** HLA-DR antigens in benign and malignant breast lesions

| *Tumour type* | *No. of cases examined* | *No. of cases classified according to % of tumour cells stained by TAL-1B5* | | | |
|---|---|---|---|---|---|
| | | *0%* | *~1%* | *2–25%* | *26–100%* |
| *Benign lesions* (*total*) | (50) | (13) | (20) | (16) | (1) |
| Fibroadenoma | 10 | – | 4 | 6 | – |
| Fibrocystic disease | 25 | 7 | 9 | 9 | – |
| Cystosarcoma phyllodes | 11 | 5 | 5 | 1 | – |
| Intraductal papilloma | 3 | 1 | 2 | – | – |
| Lactating adenoma | 1 | – | – | – | 1 |
| *Carcinomas* (*total*) | (72) | (29) | (30) | (8) | (5) |
| Infiltrating ductal ca | 39 | 16 | 17 | 3 | 3 |
| Infiltrating lobular ca | 8 | 2 | 4 | 2 | – |
| Lobular *in situ* ca | 3 | – | 2 | 1 | – |
| Medullary ca | 5 | 1 | 2 | – | 2 |
| Paget's disease | 2 | 1 | 1 | – | – |
| Papillary ca | 1 | 1 | – | – | – |
| Adenoid cystic ca | 6 | 2 | 3 | 1 | – |
| Anaplastic poorly differentiated ca | 2 | 1 | 1 | – | – |
| Male breast ca | 2 | 1 | – | 1 | – |
| Skin metastases | 4 | 4 | – | – | – |

**Figure 1** Normal resting breast epithelium is unstained while a few infiltrating mononuclear cells in the stroma express HLA-DR antigens ( x 160).

**Figure 2** Normal breast tissue from the 29th week of gestation shows homogeneous staining of the acinar epithelium as well as most of the infiltrating cells. Note the lack of HLA-DR expression in ductal epithelial cells ( x 100).

**Figure 3** Patchy HLA-DR positivity of a fibroadenoma with sparse, evenly distributed mononuclear cell infiltrate ( x 160).

**Figure 4** Almost homogeneously stained area of a fibroadenoma with relatively high density of infiltrating leukocytes ( x 100).

**Figure 5** High level of HLA-DR expression in a lactating adenoma ( x 100).

**Figure 6** HLA-DR-positive tumour cell nests of an infiltrating ductal carcinoma next to negative non-malignant epithelium ( x 100).

**Figure 7** Homogeneous positivity of staining for HLA-DR antigens in a medullary breast carcinoma ( x 160).

**Figure 8** Ductal carcinoma nodule with only rare HLA-DR-positive cells ( x 160).

mononuclear cellular infiltrate was sparse in all the above mentioned samples of the normal breast showing only few cells stained by TAL-1B5 (Figure 1).

*Differentiating mammary gland*

The immunoperoxidase staining of sections of human mammary gland tissues at different stages of physiological differentiation revealed drammatic changes of HLA-DR expression. Thus, we found nearly all acinar epithelial cells and some ductal epithelial cells positive in both late pregnancy (Figure 2) and lactation. On the other hand, the staining pattern of epithelial cells in sections of two cases of the post-weaning (regressing) breast was heterogeneous with considerably fewer positive cells than in the samples of the late-pregnant or lactating mammary gland. In comparison with the resting breast somewhat increased density of the mononuclear cell infiltrate, partly expressing HLA-DR antigens, was noted in the late-pregnant, lactating and regressing mammary gland tissue. In common with the resting gland, some mononuclear (mainly lymphoid) cells stained by TAL-1B5 were located directly within the epithelial parenchyma, usually in a 'basal' position near the basement membrane or between myoepithelial and luminal cells.

*Breast tumours*

Histopathological diagnoses and immunohistochemical staining data on sections from the samples of 50 benign and 72 malignant breast lesions are summarised in Table I.

In most benign lesions, at least a small subpopulation of epithelial cells stained with the anti-HLA-DR antibody. In the cases containing some positive cells, two distinct patterns were observed. The expression of HLA-DR antigens was usually limited to single epithelial cells or clusters of cells and an example of this focal positivity is shown in Figure 3. In some lesions, areas showing nearly homogeneous staining of the majority of epithelial cells were found (Figure 4) though the total percentage of positive epithelial cells never exceeded 25% even in such cases. The only exception among benign lesions was a lactating adenoma in which almost all cells were stained (Figure 5). Total absence of epithelial cell staining was seen in several cases of fibrocystic disease and cystosarcoma phyllodes, where only endothelial cells and infiltrating mononuclear cells appeared to express HLA-DR antigens.

Breast carcinomas exhibited greater variation with respect to the proportion of positive tumour cells than benign lesions (see Table I). Thus, although staining patterns similar to those seen in benign tumours, i.e. total negativity, patchy positivity and/or more uniform staining of some areas were observed, there was a group of cancers ($\sim$7% of the cases examined) in which more than 30% of tumour cells stained for HLA-DR (Figure 6). In 3 of these more positive cases, between 70 and 95% of the carcinoma cells appeared to be stained by the antibody against HLA-DR antigens. Among different types of breast carcinomas, medullary carcinomas were of special interest as 2 out of 5 belonged to that minor group of the highly positive cases (Figure 7). The TAL-1B5 staining reaction was both membrane-associated and cytoplasmic showing variable intensity throughout the lesions. In addition to female breast tumours, one of the two carcinomas of the male breast also revealed a subpopulation of HLA-DR-positive tumour cells.

Besides the positive cases described above, there was a considerable number of carcinomas lacking any detectable staining with the TAL-1B5 antibody, including all 4 skin metastases examined. Those malignant lesions which appeared to be almost completely unstained and showed only rare single positive cells in some tumour nodules were also classified as negative, inasmuch as it was not possible to be sure if the stained cells were infiltrating mononuclear cells (macrophages) or carcinoma cells (Figure 8). The non-malignant epithelium associated with the cancer, sometimes morphologically indistinguishable from normal, was found on sections of 32 carcinoma cases examined. In $\sim$70% of these cases, the residual non-malignant epithelium revealed focal positivity of HLA-DR antigens similar to that seen in benign breast lesions.

To confirm that the antibody TAL-1B5 was reacting with the same component in breast tumours as it was in lymphoid cells (Adams *et al.*, 1983), Western blots of gel-separated extracts of several tumours were stained with the antibody. The immunoblotting data were consistent with TAL-1B5 reacting with only one band in the region of 30–34 kDa, even in the extracts of carcinomas with a high proportion of immunohistochemically positive tumour cells accompanied by only moderately positive infiltrating leukocytes (Figure 9, lanes 1, 2 and 3).

When the density and distribution of the mononuclear cellular infiltration was evaluated, the patterns of the benign and malignant lesions showed a clear difference: almost all benign tumours contained only sparse and evenly distributed leukocytic infiltration (Figure 3), whereas carcinomas usually revealed dense infiltration (Figures 6, 7) often with large aggregates of leukocytes present at the periphery of the tumour nodules. Although occasional areas in some benign lesions were more densely infiltrated by leukocytes, especially in some fibroadenomas (Figure 4) and intraductal papillomas, the intensity of the infiltration rarely reached the level commonly seen in carcinomas. The proportion of HLA-DR-positive infiltrating leukocytes varied considerably in both benign and malignant lesions, the TAL-1B5-stained elements including some lymphoid cells, elongated cells reminiscent of dendritic cells as well as monocytes–macrophages. No obvious correlation between HLA-DR antigen expression on tumour cells and surrounding inflammatory mononuclear cells was noted.

**Figure 9** Immunoperoxidase staining of Western blots of gel-separated extracts of a medullary breast carcinoma (tracks 1–3) and cultured reduction mammoplasty organoids (tracks 4, 5) with the antibody TAL-1B5 (tracks 1, 4), BA17 (tracks 2, 5) and TF-1 (track 3).

**Figure 10** Reduction mammoplasty organoids on day 4 of collagen gel culture do not stain with the TAL-1B5 antibody (×160).

*Proliferating breast epithelium in collagen gels*

To see whether there was any correlation between the rate of proliferation and the expression of HLA-DR molecules, reduction mammoplasty organoids were cultured in collagen gels, pulsed by tritiated thymidine and sections of these organoids within the three-dimensional matrix were evaluated by combining TAL-1B5 immunohistochemistry with autoradiography on parallel sections. On day 4 of culture, sections revealed foci of organised proliferation extending from the embedded epithelial organoids into the collagenous matrix, the mean labelling index being 12.2±1.7%. From day 5 until day 10, the proliferating organoids and their newly formed processes increased in diameter and length showing mean thymidine labelling indices of 9.6±1.4% and 6.1±0.9% on day 7 and 10 of the culture period, respectively. None of the organoids at any of the three time points examined stained with the anti-HLA-DR antibody (Figure 10). The immunohistochemical data were confirmed on Western blots of gel-separated extracts from the cultured mammary organoids: No positive band was found with TAL-1B5 despite the fact that enough protein material of epithelial origin was used for this analysis as indicated by the 40 kDa cytokeratin band stained by the antibody BA17 (Figure 9, lanes 4 and 5).

### Discussion

Phenotypic changes which occur during tissue differentiation as well as during development of malignancy include those of the spectrum of cell membrane molecules, the most obvious candidates likely to be of importance in cell-cell and cell-matrix interactions. Among the membrane molecules known to be involved in cellular recognition, the class II molecules are of special interest being the products of a multigene family expressed in a differentiation-dependent or activation-dependent manner on a variety of cell types including several epithelia (see Radka *et al.*, 1986 and Forsum *et al.*, 1985, for review).

As far as mammary gland differentiation is concerned, Klareskog *et al.* (1980) reported induction of Ia antigens in guinea pig mammary gland epithelium in pregnancy and following administration of lactotropic hormones. Newman *et al.* (1980) described HLA-DR antigens on lactating human breast epithelium and isolated milk fat globule membranes suggesting that differentiation-associated changes of class II antigens similar to those found in guinea pigs (Klareskog *et al.*, 1980) might occur in humans as well but, to our knowledge, no further data on this topic have since been published. The available data on HLA-DR expression in normal resting breast epithelium include both negative (Newman *et al.*, 1980; Bernard *et al.*, 1984) and positive (Bhan & Des Marais, 1983; Natali *et al.*, 1983) reports. In the present study, we found high amounts of HLA-DR antigens on human breast epithelium at late pregnancy and lactation, whereas diminished expression and total absence of staining was observed in samples from the regressing (post weaning) period and in the normal resting breast, respectively. We believe this is the first report concerning the expression of HLA-DR antigens at all important stages of human breast differentiation and our results support the notion that the changes follow in humans a similar pattern to that in guinea pigs (Klareskog *et al.*, 1980; and this report). Furthermore, our findings on the absence of HLA-DR molecules in normal resting breast epithelium obtained from reduction mammoplasties and from apparently healthy women killed in road accidents, as opposed to occasional patchy positivity of the TAL-1B5 antibody staining on 'normal' breast epithelium obtained from mastectomies, provide a possible explanation of the conflicting results reported by various investigators for normal mammary gland epithelium. Thus, the authors who reported the lack of staining for HLA-DR antigens used reduction mammoplasty as their material source (Newman *et al.*, 1980; Bernard *et al.*, 1984) while in both positive reports (Bhan and Des Marais, 1983; Natali *et al.*, 1983), the uninvolved 'normal' tissues removed with tumours at mastectomy were used. Although the transient patchy expression of class II molecules in the normal human resting breast epithelium, e.g. at a certain stage in the menstrual cycle, cannot be excluded at present, our data together with those published by the above mentioned authors rather suggest occasional aberrant HLA-DR expression on the 'normal' epithelium of the cancerous breasts, thus providing another warning against the use of uninvolved mastectomy tissues as controls in the studies of various aspects of breast cancer biology.

In contrast to Bernard *et al.* (1984) who reported no staining with an anti-HLA-DR monoclonal antibody on any of the 10 fibroadenomas examined, we found the majority of the benign breast lesions heterogeneously positive, the top values for the stained epithelial cells being about 15–25%. The exceptional case of the lactating adenoma revealed almost homogeneous positivity with TAL-1B5, thus resembling the phenotype of the lactating breast epithelium. Our immunohistochemical study of 50 cases of the benign breast lesions generally confirmed and somewhat extended the findings by Bhan and Des Marais (1983) and Whitwell *et al.* (1984) of the heterogeneous expression of HLA-DR antigens by a proportion of the epithelial cells in the benign human mammary tumours.

The results of our present study based on the examination of the large panel of breast carcinomas do not support the notion that the expression of HLA-DR antigens by the malignant tumour cells is a rare (Bhan & Des Marais, 1983) or even non-existent (Hurliman & Saraga, 1985) event. The variability of staining for HLA-DR on the breast carcinoma cells reported here is consistent with previous immunohistochemical data published by Natali *et al.* (1983), Whitwell *et al.* (1984) and Göttlinger *et al.* (1985). The use of paraffin-embedded tissue sections which provide superior preservation of morphologic features, rather than frozen sections employed by the investigators referred to above, permitted more definitive analysis, a factor that becomes critical in the attempt to discriminate between a wholly negative tissue or one which contains some scattered positive carcinoma cells. The lower resolution limits of cryostat sections together with their commonly smaller size and the general lack of any precise criteria by which a tumour might be classified as positive represent just a few out of the many factors potentially responsible for the discrepancies reported by different authors. To exclude the possibility that the anti-HLA-DR antibody used in our study detected some cross-reacting epitope of a molecule present on the breast tumour cells, in addition to HLA-DR antigens present on infiltrating leukocytes, we deployed the immunoblotting technique with tumour lysates separated by SDS-PAGE. The fact that TAL-

1B5 always revealed only the band predicted for the HLA-DR polypeptides supports our conclusion that the positive staining was due to genuine HLA-DR antigens. One observation of potential importance is the existence of a small group of breast carcinomas expressing high levels of HLA-DR molecules (this study, Göttlinger *et al*., 1985; Bhan & Des Marais, 1983) and future efforts correlating clinical parameters of breast carcinomas with their HLA-DR phenotype may be warranted.

The mechanisms which regulate the expression of class II molecules on mammary gland epithelial cells are poorly understood. Recent evidence suggests that recombinant human gamma interferon, an inducer of HLA-DR expression in competent immune cells, induces the synthesis of HLA-DR molecules by various human breast cancer cell lines *in vitro* (Gastl *et al*., 1985). The expression of class II antigens in guinea pig mammary gland epithelial cells is induced by pregnancy and lactation and can also be induced by exogeneous administration of lactotropic hormones (Klareskog *et al*., 1980) suggesting hormonal regulation of this phenomenon. In a similar fashion, the data of Newman *et al*. (1980) extended by our present results demonstrate reversible pregnancy-associated induction of HLA-DR antigens on normal human breast epithelium. Furthermore, recent experiments of Bernard *et al*. (1986) have shown that prolactin added to culture medium increases class II antigenic expression by MCF-7 breast cancer cell line. Increased levels of HLA-DR antigens on epithelial cells in late pregnancy, lactation as well as in some breast tumours, i.e. conditions known to be associated with higher proliferation rate, suggested to us a possible relationship between the increased mitotic rate and the induction of HLA-DR molecules on human mammary gland epithelial cells. In this context, it is interesting that the expression of HLA-DR antigens in endometrial epithelium was reported to parallel the rise and fall of DNA synthesis and mitoses (Tabibzadeh *et al*., 1986) though this correlation was not found in a previous study by Ferguson *et al*. (1985). To test the hypothetical association of the HLA-DR expression with proliferation, we deployed the collagen gel culture of breast epithelial organoids pulsed with tritiated thymidine followed by the combination of immunohistochemical staining for HLA-DR and autoradiography. We believe the analysis of this *in vitro* model demonstrates for the first time that proliferation itself, even at a high rate, is not sufficient for the induction of class II antigens on normal human mammary gland epithelium. This conclusion is in accordance with the findings by Gastl *et al*. (1985) that HLA-DR expression induced by gamma interferon in several breast cancer cell lines did not depend on proliferation but required intact RNA and protein synthesis.

It is presently far from clear what the biological role of class II molecules on either epithelial cells or carcinomas is. Speculations concerning normal mammary gland epithelium include e.g. involvement of epithelial class II antigens in recruitment of lymphoid cells in lactation as a part of the enteromammary pathway of protective immunity to the offspring (Klareskog *et al*., 1980; Forsum *et al*., 1985), and a role in providing a system for transporting key intracellular peptides to the extracellular milieu was suggested by Unanue and Allen (1986) for epithelia involved in peptide transport in general. Class II molecules on epithelial cell membranes may be involved in antigen presentation to T-cells, as has been suggested for thyroid epithelium which is capable of expressing HLA-DR molecules and presenting antigens to cloned human T lymphocytes (Londei *et al*., 1984). The latter idea is particularly attractive in view of the expression of HLA-DR antigens by some carcinomas including breast tumours which could have implications for the induction of immune responses to putative tumour-specific or tumour-associated antigens. Further studies are required to test these and other hypotheses.

We wish to thank Sir Walter Bodmer of the Imperial Cancer Research Fund, London, for supplying the monoclonal antibody TAL-1B5, and Dr R.R. Millis of the Hedley Atkins Unit, Guy's Hospital, London, for contributing the paraffin sections of the late-pregnant human breast tissue and the lactating adenoma. The excellent technical assistance of Mrs J. Hanyková, Mrs L. Hladká, Mrs T. Kamenická and Mrs J. Ševčiková is also gratefully acknowledged.

### References

ADAMS, T.E., BODMER, J.G. & BODMER, W.F. (1983). Production and characterization of monoclonal antibodies recognizing the α-chain subunits of human Ia alloantigens. *Immunology*, **50**, 613.

BÁRTEK, J., VIKLICKÝ, V., FRANĚK, F. & 5 others (1982). Monoclonal antibodies against transferrin. Precipitating mixtures and lack of inter-species cross-reactivity. *Immunol. Lett*., **4**, 231.

BÁRTEK, J., DURBAN, E.M., HALLOWES, R.C. & TAYLOR-PAPADIMITRIOU, J. (1985*a*). A subclass of luminal epithelial cells in the human mammary gland defined by antibodies to cytokeratins. *J. Cell Sci*., **75**, 1.

BÁRTEK, J., TAYLOR-PAPADIMITRIOU, J., MILLER, N. & MILLIS, R. (1985*b*). Patterns of expression of keratin 19 as detected with monoclonal antibodies in human breast tissues and tumours. *Int. J. Cancer*, **36**, 299.

BENACERRAF, B. (1985). Significance and biological function of class II MHC molecules. *Amer. J. Pathol*., **120**, 334.

BERNARD, D., MAURIZIS, J.-C., CHASSAGNE, J., CHOLLET, P. & PLAGUE, R. (1986). Effect of prolactin on class II HLA antigen expression by MCF-7 cell line. *Anticancer Res*., **6**, 79.

BERNARD, D.J., MAURIZIS, J.-C., RUSÉ, F. & 5 others (1984). Presence of HLA-D/DR antigens on the membrane of breast tumour cells. *Clin. Exp. Immunol*., **56**, 215.

BHAN, A.K. & DES MARAIS, C.L. (1983). Immunohistologic characterization of major histocompatibility antigens and inflammatory cellular infiltrate in human breast cancer. *J. Natl Cancer Inst*., **71**, 507.

BODMER, J. & BODMER, W. (1984). Histocompatibility 1984. *Immunology Today*, **5**, 251.

BURCHELL, J., DURBIN, H. & TAYLOR-PAPADIMITRIOU, J. (1983). Complexity of expression of antigenic determinants, recognized by monoclonal antibodies HMFG-1 and HMFG-2 in normal and malignant human mammary epithelial cells. *J. Immunol*., **131**, 508.

DURBAN, E.M., BUTEL, J.S., BÁRTEK, J. & TAYLOR-PAPADIMITRIOU, J. (1986). The importance of matrix interactions and tissue topography for the growth and differentiation of mammary cells *in vitro*. In *Breast Cancer: Origins, Detection and Treatment*, Rich, M.A. *et al*., (eds) p. 13. Martinus Nijhoff Publishing: Boston.

EMERMAN, J.T. & PITELKA, D.R. (1977). Maintenance and induction of morphological differentiation in dissociated mammary epithelium on floating collagen membranes. *In Vitro*, **13**, 316.

EPENETOS, A.A., BOBROW, L.G., ADAMS, T.E., COLLINS, C.M., ISAACSON, P.G. & BODMER, W.F. (1985). A monoclonal antibody that detects HLA-DR region antigen in routinely fixed, wax embedded sections of normal and neoplastic lymphoid tissues. *J. Clin. Pathol*., **38**, 12.

FERGUSON, A., MOORE, M. & FOX, H. (1985). Expression of MHC products and leucocyte differentiation antigens in gynaecological neoplasms: An immunohistochemical analysis of the tumour cells and infiltrating leucocytes. *Br. J. Cancer*, **52**, 551.

FORSUM, U., CLAESSON, K., HJELM, E. & 4 others (1985). Class II transplantation antigens: Distribution in tissues and involvement in disease. *Scand. J. Immunol*., **21**, 389.

GASTL, G., MARTH, C., LEITER, E. & 5 others (1985). Effects of human recombinant $\alpha_2$ arg-interferon and $\gamma$-interferon on human breast cancer cell lines: Dissociation of antiproliferative activity and induction of HLA-DR antigen expression. *Cancer Res.*, **45**, 2957.

GÖTTLINGER, H.G., RIEBER, P., GOKEL, J.M., LOHE, K.J. & RIETHMÜLLER, G. (1985). Infiltrating mononuclear cells in human breast carcinoma: Predominance of $T4^+$ monocytic cells in the tumour stroma. *Int. J. Cancer*, **35**, 199.

HURLIMAN, J. & SARAGA, P. (1985). Mononuclear cells infiltrating human mammary carcinomas: Immunohistochemical analysis with monoclonal antibodies. *Int. J. Cancer*, **35**, 753.

KAUFMAN, J.F., AUFFRAY, C., KORMAN, A.J., SHACKELFORD, D.A. & STROMINGER, J. (1984). The class II molecules of the human and murine major histocompatibility complex. *Cell*, **36**, 1.

KLARESKOG, L., FORSUM, U. & PETERSON, P.A. (1985). Hormonal regulation of the expression of Ia antigens on mammary gland epithelium. *Eur. J. Immunol.*, **10**, 958.

LONDEI, M., LAMB, J.R., BOTTAZZO, G.F. & FELDMAN, M. (1984). Epithelial cells expressing aberrant MHC class II determinants can present antigen to cloned human T-cells. *Nature*, **312**, 639.

NATALI, P.G., GIACOMINI, P., BIGOTTI, A. & 4 others (1983). Heterogeneity in the expression of HLA and tumour-associated antigens by surgically removed and cultured breast carcinoma cells. *Cancer Res.*, **43**, 660.

NEWMAN, R.A., ORMEROD, M.G. & GREAVES, M.F. (1980). The presence of HLA-DR antigens on lactating human breast epithelium and milk fat globule membranes. *Clin. Exp. Immunol.*, **41**, 478.

RADKA, S.F., CHARRON, D.J. & BRODSKY, F.M. (1986). Review: Class II molecules of the major histocompatibility complex considered as differentiation markers. *Human Immunol.*, **16**, 390.

STAMPFER, M., HALLOWES, R.C. & HACKETT, A.J. (1980). Growth of normal human mammary cells in culture. *In Vitro*, **16**, 415.

TABIBZADEH, S.S., BETTICA, A. & GERBER, M.A. (1986). Variable expression of Ia antigens in human endometrium and in chronic endometritis. *Amer. J. Pathol.*, **86**, 153.

UNANUE, E.R. & ALLEN, P.M. (1986). Comment on the finding of Ia expression in nonlymphoid cells. *Lab. Invest.*, **55**, 123.

WHITWELL, H.L., HUGHES, H.P.A., MOORE, M. & AHMED, A. (1984). Expression of major histocompatibility antigens and leucocyte infiltration in benign and malignant human breast disease. *Br. J. Cancer*, **49**, 161.